# Artificial Intelligence for Clinical Decision Support in Sepsis

**DOI:** 10.3389/fmed.2021.665464

**Published:** 2021-05-13

**Authors:** Miao Wu, Xianjin Du, Raymond Gu, Jie Wei

**Affiliations:** ^1^Department of Emergency, Renmin Hospital of Wuhan University, Wuhan, China; ^2^Department of Critical Care Medicine, Renmin Hospital of Wuhan University, Wuhan, China; ^3^Department of Surgery, State University of New York Upstate Medical University, Syracuse, NY, United States

**Keywords:** sepsis, artificial intelligence, machine learning, deep learning, early prediction

## Abstract

Sepsis is one of the main causes of death in critically ill patients. Despite the continuous development of medical technology in recent years, its morbidity and mortality are still high. This is mainly related to the delay in starting treatment and non-adherence of clinical guidelines. Artificial intelligence (AI) is an evolving field in medicine, which has been used to develop a variety of innovative Clinical Decision Support Systems. It has shown great potential in predicting the clinical condition of patients and assisting in clinical decision-making. AI-derived algorithms can be applied to multiple stages of sepsis, such as early prediction, prognosis assessment, mortality prediction, and optimal management. This review describes the latest literature on AI for clinical decision support in sepsis, and outlines the application of AI in the prediction, diagnosis, subphenotyping, prognosis assessment, and clinical management of sepsis. In addition, we discussed the challenges of implementing and accepting this non-traditional methodology for clinical purposes.

## Introduction

Sepsis is a syndrome in which infection causes host response imbalance. It leads to life-threatening organ damage, and has a high mortality rate. Sepsis not only threatens human health, but also brings a huge economic burden to medical and health care (1). Given that sepsis has a certain morbidity and high mortality, early prediction and intervention of sepsis is of great significance (2). The management of sepsis is a highly complex and challenging problem, and it is still the subject of well-trained and highly skilled experts. More than a quarter century of research has not produced a reliable diagnostic test or a direct treatment for sepsis. The core of this deficiency is that sepsis is still a clinical/physiological diagnosis, representing many molecularly different pathological trajectories. But as the applications of AI in the medical field continue to emerge, some medical decisions will soon be left to so called “intelligence” machines to improve clinical practice and patient prognosis. In fact, many tasks involved in the clinical management of sepsis can be performed individually or optimized through dedicated algorithms, including early prediction, improvement of antibiotic therapy, and hemodynamic optimization (3, 4). At present, thanks to the dissemination of electronic health records (EHR), the application of AI has a good foundation.

## Artificial Intelligence and Sepsis

In 1956, a gathering at the Dartmouth Conference proposed the concept of “artificial intelligence,” hoping to use recently developed computers to construct complex machines with the same essential characteristics as human intelligence. However, due to constraints in memory and a lack of processing power, developments in AI proceeded slowly. After 2012, thanks to the increase in data volume, computing power, and the development of new machine learning algorithms, AI began to explode, resulting in expansions in expert systems, machine learning, evolutionary computing, computer vision, natural language processing and other data processing technologies (5). Among them, mechanical learning is the most widely used in sepsis.

**Figure 1:**
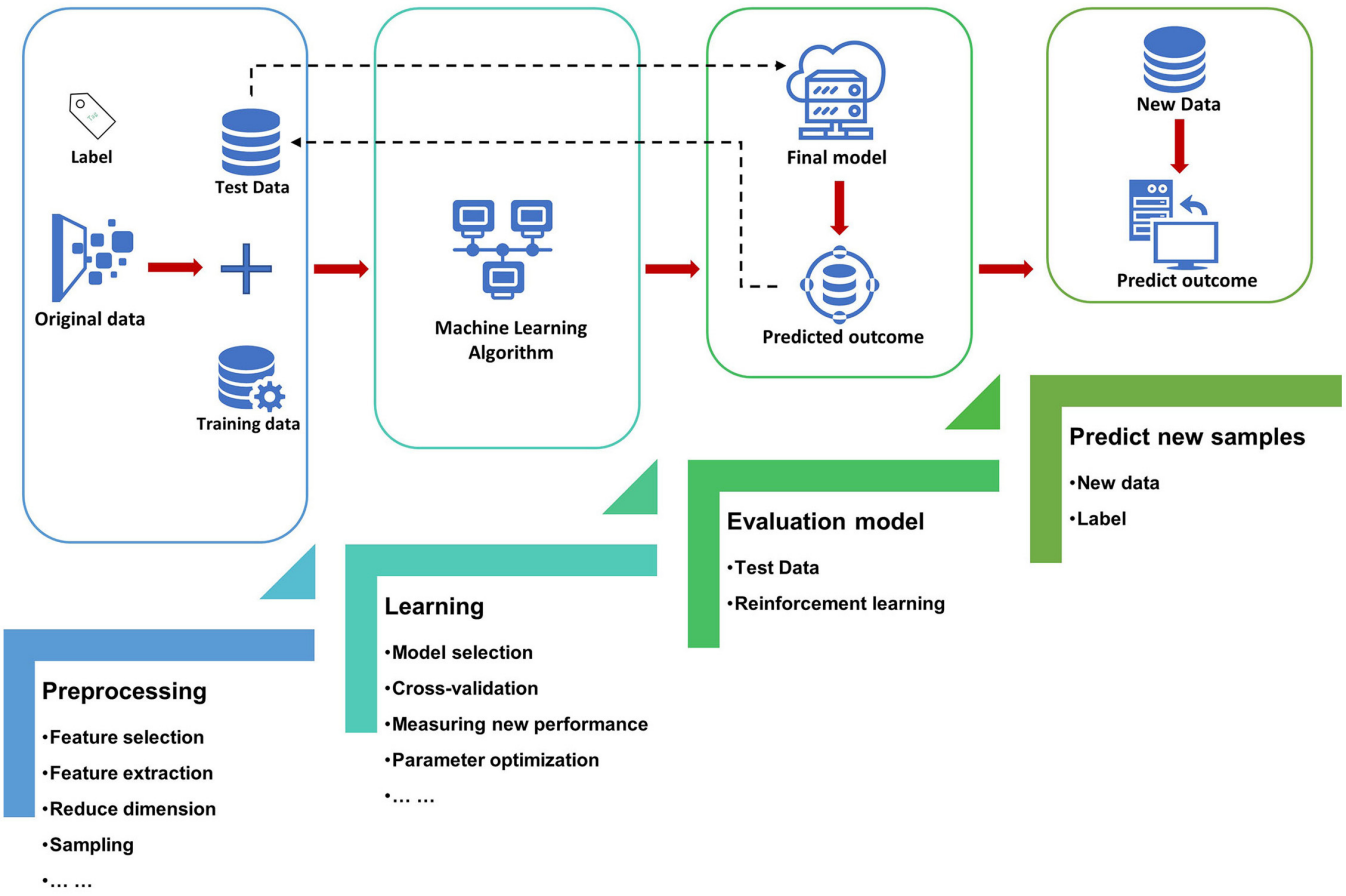
Roadmap for machine learning systems.

**Figure 2:**
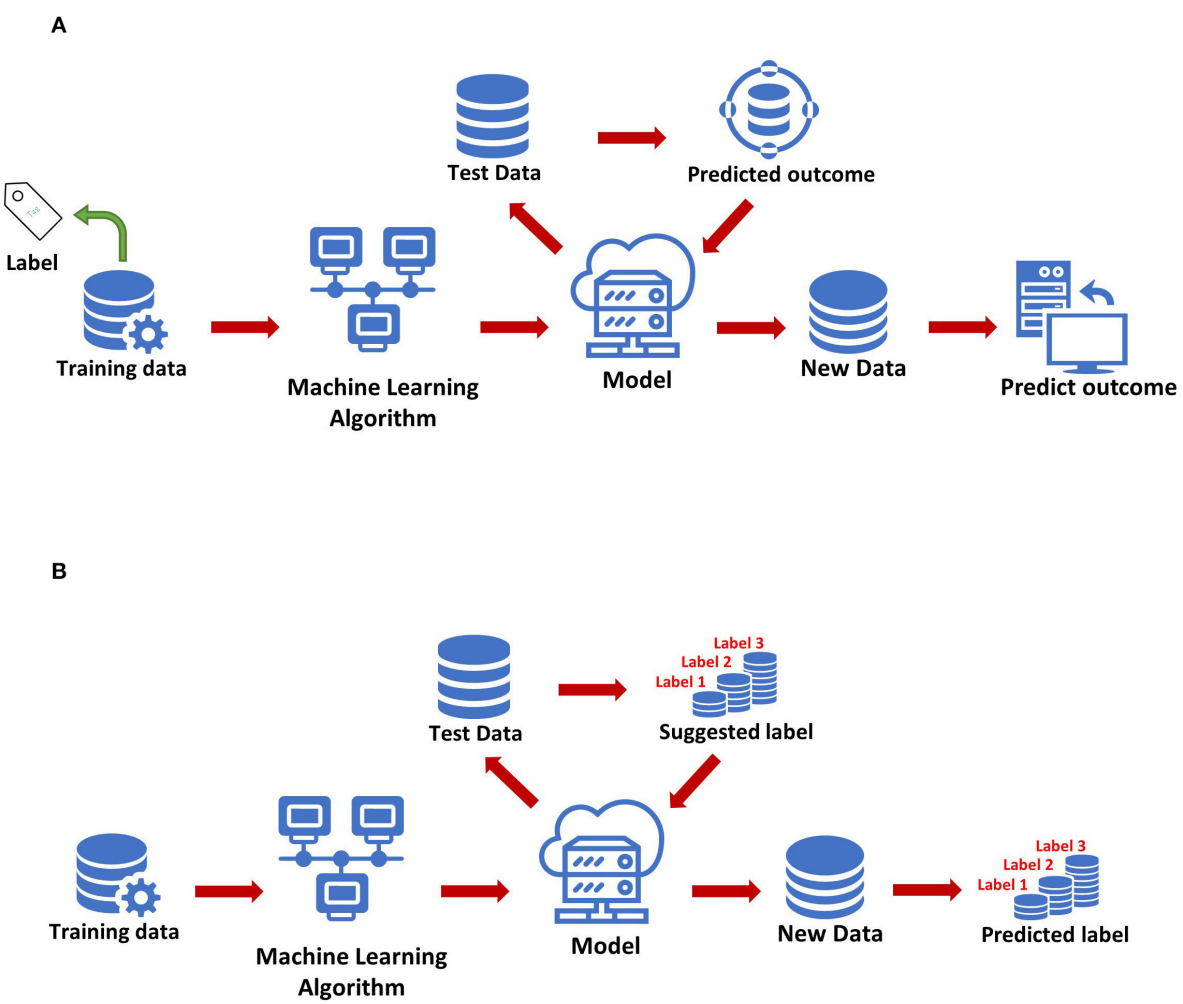
Two methods of machine learning. **(A)** Supervised learning. **(B)** Unsupervised learning.

The most basic method of machine learning uses algorithms to analyze and learn from data, and then uses the results of learning to make decisions and predictions about events in reality. Unlike traditional hard-coded software programs, machine learning uses numerous amounts of data to learn how to out specific tasks from the data using various algorithms (4) (Figure 1). Machine learning has appeared in the early stages of AI development. The initial algorithms include decision trees, support vector machine (SVM), clustering and so on. Machine learning can be classified according to different learning methods. The initial algorithms included supervised learning, unsupervised learning, and semi-supervised learning (Figure 2). Later, more algorithms such as integrated learning, deep learning, and reinforcement learning were developed. The application of traditional machine learning algorithms in sepsis management has had preliminary results, but every step forward was extremely difficult, until the emergence of deep learning.

At the very beginning, deep learning was not a brand new learning method, but a deep neural network that could be developed using supervised and unsupervised learning methods. However, due to rapid growth in the field of machine learning in recent years, some unique learning methods have been proposed (such as residual networks). As a result, more and more people regard it as a learning method alone. Originally deep learning used deep neural networks to solve feature expression. Deep neural network itself was not a new concept but could be simply understood as a neural network structure containing multiple hidden layers. People could adjust the connection and activation methods of neurons accordingly to improve the training effect of deep neural networks. In fact, there were many such ideas in the early years, but due to insufficient training data and backward calculation ability, the results were not satisfactory. Deep learning has accomplished various tasks in healthcare, including the management of sepsis (6–12), which provides the possibility for its application in clinical practice.

## Methods of Literature Selection

The literature search was conducted in (PubMed). Research papers, systematic reviews, and narrative reviews published prior to January 31, 2021 were included. Abstracts without full text were excluded. The search terms used to find relevant literature included: (“machine learning” OR “deep learning” OR “neural network” OR “artificial intelligence”) AND (“sepsis”). A total of 433 papers were initially identified with these search terms, of which 33 abstracts without full text were excluded, leading to a final count of 400. Given the narrative nature of this review, the final cohort of papers was hand-picked to provide the reader with the best general overview of the topic and was not meant to be comprehensive. We selected some research manuscripts and systematic reviews, and referenced a number of narrative reviews. This article is based on previously conducted studies and does not contain any studies with human participants or animals performed by any of the authors.

## Application of AI in the Early Prediction and Diagnosis of Sepsis

### Early Prediction of Sepsis

Early intervention of sepsis is the key to treatment, as every hour of delay in treatment increases mortality. If we can predict the occurrence of sepsis early, we can initiate intervention measures as soon as possible. The original sepsis prediction system relies mainly on empirical clinical decision rules (CDR), which usually uses vital signs collected at the bedside. For example, five physiological markers are extracted from the bedside monitor every minute. These data streams include heart rate, respiratory rate, and blood pressure (systolic, diastolic, and mean blood pressure), and then are classified by SVM classifiers (13). The model can accurately predict the incidence of sepsis, with an average detection accuracy of 83.0% and an Area Under Receiving Operator Characteristics (AUROC) of 0.781. This is the minimal AI model developed for early prediction of sepsis. Logistic regression is also used to measure six variables related to sepsis, and a predictive model (automated screening tool) with an AUROC of 0.857 has been developed to help identify patients at risk of sepsis (14). The screening tool can screen all hospitalized patients and pass the results directly to caregivers without any manual intervention.

The main disadvantage of CDR is that when used in a population different from the derived population, there will be generality and performance differences. In addition, it usually takes several years to establish and verify. The growth of deep learning has created more opportunities for the application of AI in sepsis (15–18). Bi-Directional Gated Recurrent Units (GRU) is a deep learning algorithm that uses various parameters related to the vitals, laboratory, and demographics (6). The AUROC of this model is 0.97, which can predict the occurrence of sepsis 6 h in advance. This method is better than the AI models for sepsis prediction found in the current literature. There is also an early warning system for sepsis using deep learning. A new algorithm based on electronic medical record (EMR) was designed, which can detect sepsis 6 h before the occurrence of sepsis, with an AUROC of 0.782 (7). Another sepsis detection system uses a convolutional neural network and a long short-term memory network (12). The quality evaluation of the model is based on standard concepts of accuracy and clinical applicability, and the intervention is evaluated retrospectively by observing intravenous antibiotics and blood cultures before the predicted time. The AUROC at 3 h before the onset of sepsis was 0.856. In the past, due to the delay in sepsis recognition, vast majority of sepsis patients did not start antibiotic treatment or blood culture in time. Therefore, this model can promptly facilitate such interventions through early identification.

With the progress of deep learning, more and more studies have introduced it into clinical decision support for sepsis. In order to evaluate its function, the performance of deep learning was compared to other methods in the early prediction of sepsis, including three machine learning algorithms (random forest, Cox regression and penalized logistic regression) and three scoring screening tools (SIRS, qSOFA and NEWS) (9). Demographics, comorbidities, vital signs, medicines, and test results are all included in the training data set. Multi-output Gaussian process and recurrent neural network (MGP-RNN), a deep learning-based model that can advance the prediction of sepsis by 5 h, performed the best.

In addition to the above-mentioned deep learning, some people have developed an explainable AI model for early prediction of sepsis. They developed a model based on shared ICU public data and verified the challenge score in a completely hidden population (19). The explainable AI model extracts 168 features per hour and is trained to achieve real-time prediction of sepsis. The influence of each feature on the real-time prediction of sepsis is discussed in depth to show its interpretability. This model not only has superior performance in estimating the risk of sepsis in real time, but also provides interpretable information for comprehending the risk of sepsis.

However, traditional supervised models tend to perform better only in certain aspects compared to ensemble learning. Ensemble learning is a comprehensive strongly supervised model, usually composed of multiple weakly supervised models. The potential goal of ensemble learning is that even if one of the weak classifiers makes a prediction error, the other weak classifiers can correct the error. Recently, a study reported a sepsis prediction model based on ensemble learning framework, which combines artificial features extracted from advanced clinical knowledge and deep features based on automatic extraction of long-term and short-term memory (LSTM) neural networks (20). Through ensemble learning, the early prediction of sepsis was achieved 6 h in advance. The results show that the model has a good effect on early detection of sepsis. In particular, ensemble learning is significantly better than other single models in performance (Table 1).

**Table 1:** Summary of the results from related works on the prediction of sepsis onset.

**Authorship**	**Year**	**Subjects**	**Features**	**Techniques**	**Best model/Algorithms**	**AUROC**	**References**
Misra et al.	2021	45,425	15	• Apache Spark • random under-sampling algorithm • 5-fold cross validation	Random Forest	0.9483	(21)
Wardi et al.	2021	183,573	40	• transfer learning • a modified Weibull-Cox proportional hazards model • optimized using gradient descent	Artificial Intelligence Sepsis Expert	0.833	(22)
Wickramaratne et al.	2020	40,336	36	• Recurrent Neural Network Variant • 5% recurrent dropout and early stopping schemes • Nesterov Adam optimizer	Bi-Directional Gated Recurrent Units	0.97	(6)
Lee et al.	2020	60,000	40	• deep learning-based early warning system • score function used in the Physionet Challenge 2019 • Noisy Data Imputation	Graph Convolutional Network	0.782	(7)
Kok et al.	2020	2,932	40	• Gaussian Process Regression • Radial Basis Function kernel combined with White Noise kernel • 10-fold cross validation	Temporal Convolution Network	0.98	(8)
Bedoya et al.	2020	42,979	86	• variety of imputation strategies • Internal validation • Temporal validation	Multi-output Gaussian Process and Recurrent Neural Network	0.88	(9)
Lauritsen et al.	2020	52,229	30	• 5-fold cross validation • Gradient Boosting Classifier • multilayer feedforward neural network	Convolutional Neural Network and Long Short-term Memory Network	0.856	(12)
Mohammed et al.	2020	5,958	5	• physiological data streams	Support Vector Machine	0.781	(13)
Cooper et al.	2020	10,792	6	• Logistic regression	Automated Sepsis Screening Tool	0.857	(14)
Helguera-Repetto et al.	2020	236	25	• SupplementaryMaterial • 5-fold-cross-validation • Internal Validation (Slope and Intercept Test)	Artificial Neural Network	0.944	(23)
Kaji et al.	2020	56,841	119	• Philippe Re'my's Github repository • a TensorFlow backend • RMSProp optimizer	Long Short-Term Memory Recurrent Neural Network	0.876	(16)
Yuan et al.	2020	1,588	106	• TED_ICU (continuous data recording) • 5-fold cross-validation • a decision-tree based algorithm	XGBoost	0.89	(24)
Bloch et al.	2019	4,534	4	• Support Vector Machine with radial basis function • 10-fold cross validation • features which represent the variability in vital signs	Support Vector Machine	0.8838	(25)
Scherpf et al.	2019	46,520	10	• 4-fold-stratified-cross-validation • Gated recurrent unit • optimized on binary cross-entropy cost function	Recurrent Neural Network	0.81	(17)
Liu et al.	2019	38,645	128	• Natural Language Processing features • GloVe/GRU-based method • a gradient boosting model	XGBoost	0.92	(26)

### Early Prediction of Septic Shock

The development of decision support systems that relied on advances in machine learning is a field of innovation in healthcare strategies. Predicting the development of septic shock is one of the active areas (27). Many studies have developed intelligent decision support tools related to septic shock to improve clinical results and promote real-time optimization of medical resources. One of the studies compared eight different machine learning algorithms with the goal of developing a predictive model of septic shock, including Random Forest, C5.0, Decision Trees, Boosted Logistic Regression, SVM, Logistic Regression, Regularized Logistic, and Bayes Generalized Linear Model (21). The model using the Random Forest algorithm performed best, with an AUROC of 0.9483, a sensitivity of 83.9%, and a specificity of 88.1%. There are also studies using gradient enhancement algorithms to develop septic shock prediction models, such as XG-Boost, by combining physiological data in EHR with features obtained from natural language processing in clinical medical record data. Among them, the median warning time of the best method is 7.0 h, which is enough to intervene many hours before the onset of septic shock (26).

Transfer learning is a new subfield of machine learning, which allows the promotion of algorithms in various clinical sites. In order to study the effectiveness of AI Sepsis Expert in predicting delayed septic shock in ED, transfer learning was introduced, and the feasibility of improving external effectiveness in the second location was verified (22). The best AUROC of this AI is <0.8, and it has the best performance in predicting delayed septic shock at 8 and 12 h. Transfer learning greatly improves the external validity and generality of the model.

### Improve the Accuracy of Sepsis Diagnosis

Multiple organ failure is a typical manifestation of sepsis and is closely related to the diagnosis of sepsis. However, multiple organ failure itself often has no typical clinical manifestations, which aggravates the complexity of sepsis diagnosis and affects the accuracy of diagnosis. In order to solve the dilemma of the current diagnosis of sepsis, some studies have developed affordable automated diagnostic tools (28). Kok et al. developed a deep temporal convolution network model for sepsis detection, and evaluated it through three verification methods. The final selected model was robust and can be used as an early diagnosis tool for sepsis in the hospital. The accuracy and precision of this diagnostic tool was relatively higher than other algorithms (8). Another study introduced the development of an AI algorithm that can be used for sepsis diagnosis, and compares its performance with the diagnostic method based on SOFA score (24). The algorithm used pre-selected features and prospectively selected 106 clinical features for sepsis diagnosis. The de-identified data was used to develop this AI. The 5-fold cross-validation was applied to assess the performance of several machine learning methods, and finally the best-performing XGBoost based on the decision tree was used in the development of the AI algorithm. The AUROC of the established AI algorithm is about 0.89, while the SOFA score is only 0.596. This AI algorithm was developed through pre-selected features and XGBoost based on data collected by EMR from real cases of sepsis patients. The accuracy of early diagnosis of sepsis exceeds 80%. The timely and accurate response of this AI algorithm can enable clinicians to deploy appropriate treatment methods earlier, which will result in lower medical costs and improved patient prognosis, so the healthcare system, medical staff and patients can all benefit from it.

However, because of the non-specific signs and symptoms, the diagnosis of neonatal sepsis remains a challenge. Traditional scoring systems help distinguish patients with sepsis from those with non-sepsis, but they did not consider the particularity of each patient. There is a neonatal sepsis model based on the training and verification of artificial neural network (ANN) algorithms, mainly for the diagnosis of early-onset and late-onset neonatal sepsis (23). The results show that compared with doctors based on the traditional scoring system, the performance of the model is superior by using the same features. The sensitivity is 93.3%, the specificity is 80.0%, and the AUROC is 94.4%. The 10 most critical factors for the evaluation of neonatal sepsis are maternal age, cervicovaginitis and neonates, fever, apnea, platelet count, gender, bradypnea, band cell, catheter use, and birth weight.

## Application of AI in the Prognosis and Risk Assessment of Sepsis

Sepsis is a relatively common cause of death in patients with suspected infection. Its current mortality rate is still high and unacceptable. Appropriate assessment tools that can be used to evaluate the prognosis of sepsis may improve the accuracy of clinical decision-making and reduce mortality (25). A deep neural network (DNN) model developed using LSTM can evaluate the clinical status of patients after treatment in the intensive care unit (ICU), thereby predicting the mortality rate within 96 h after admission. The AUC of the multi-center study was 0.88, and the AUC of the single-center study was 0.85 (10). This LSTM-based model could assist doctors identify patients with poor prognosis early, so as to “re-triage” and adjust treatment plans.

The clinical manifestations and prognosis of sepsis-associated acute kidney injury (AKI) are not all the same. AI can be used to divide them into various sub-phenotypes according to the degree of risk, thereby helping to improve the management of related patients (29). A study used deep learning to determine the subphenotype of sepsis-related AKI and predict the 28-day mortality and dialysis needs of sepsis-related AKI (30). The study utilized the K-means algorithm and used more than 2,500 feature combinations to cluster patients with sepsis-related AKI and identified three subphenotypes. Among them, subtype 1 has the lowest dialysis requirement (4%), and the 28-day mortality rate after AKI is also the lowest (23%). After adjustment, the mortality rate of subtype 3 is 1.9 times that of subtype 1. Similarly, Ibrahim et al. also used AI to stratify the types of organ dysfunction observed in patients with sepsis in the ICU, and identified clinically meaningful sepsis subgroups with different organ dysfunction patterns (31). Random forests, gradient boost trees, and SVMs are used for classification.

Coagulation disorders caused by sepsis have a poor prognosis, and there are currently no definitive tools to predict it. Machine learning technology can be used to create predictive models of coagulopathy progression. According to Japan's Septic Disseminated Intravascular Coagulation (DIC) retrospective research, machine learning algorithms including multiple linear regression (MLR), random forest, SVM and neural network were utilized to estimate the progression of coagulopathy and compare its accuracy with traditional methods (32). In terms of DIC progress, random forest has the highest prediction accuracy rate of 67.0%, and the difference between the ΔDIC predicted by random forest and real ΔDIC is 1.54, which is the smallest.

In order to predict the mortality of patients with suspected infection or sepsis in ED, the performance of AI was also been evaluated. A study compared the effects of several AIs in the classification and mortality prediction of sepsis patients in ED (33). A total of four supervised learning models, random forest, C4.5 decision tree, SVM and ANN were compared. The result is that SVW and ANN using physiological variables have the best discrimination effect. It has good application prospects in assessing the classification and prognosis of sepsis. Convolutional Neural Network plus SoftMax, a deep learning-based algorithm, can also be used to predict the mortality of patients suspected of infection in ED. The results show that compared with other machine learning algorithms and sepsis scoring tools commonly used in clinical practice (SIRS and qSOFA), the accuracy of this deep learning method is significantly superior (34). Deep learning can effectively help identify critically ill patients earlier.

## Application of AI in the Management of Patients With Sepsis

Passive leg lift (PLR) can predict fluid responsiveness in sepsis, but the patient's limited mobility usually precludes the use of this hemodynamic challenge. To predict the fluid responsiveness of patients with sepsis or septic shock, machine learning using data from transthoracic echocardiography (TTE) was developed (35). The results show that the partial least-squares regression (PLS) model has an AUC value of 0.97, which was the best model and was comparable to the hemodynamic response of PLR. The key parameters of echocardiography include inferior vena cava collapsibility, velocity-time integral, S-wave, E/Ea ratio, and E-wave. Another study also reported on fluid management strategies for patients with sepsis. Causal inference technology is used to estimate the mortality outcome caused by the “caps” setting of fluid volume administration in the first 24 h in ICU (36). It was found that if the total amount of fluid in these patients is limited to 6–10 L, the 30-day mortality rate may be lower than the mortality rate observed in current practice. The mortality rate of 8 L was found to have the largest decrease.

Sepsis bundles designed to reduce the deleterious effect of sepsis have been recommended for nearly a decade. Despite this, the mortality rate of sepsis is still high, and the compliance of sepsis bundles is still not ideal. A multidisciplinary project used the Model Cell mental model to analyze collected mortality and compliance data, and compared the observed mortality data with predicted data based on comparable acute care facilities (37). The results showed that as the bundle compliance increased, the mortality rate of the entire system decreased significantly. In the linear model, compliance alone can explain nearly two-thirds of the variance. When using only the final 12 months of the project, the median death rate dropped further to 5.3%. The Model Cell intervention successfully improved bundle compliance, thereby reducing mortality. As technology advances, this model can be enhanced and ready for AI to help drive further success.

The etiology of sepsis is also very important for the formulation of treatment strategies. Inflammatix-bacterial-viral-non-infected-version 1 (IMX-BVN-1) is a neural-network classifier that can provide an assessment tool for suspected infected patients on admission (38). It can improve the recognition of bacterial and viral infections, reduce the overuse of antibiotics, block the progression of sepsis, and cut down the healthcare costs.

Critically ill patients in the ICU have an increased risk of infection due to their unique physiological changes, and various special pathogens in the environment can also increase their mortality. Due to various issues, the dosage of antibacterial agents in the ICU may become a tricky matter. These difficulties make the standard antimicrobial dosage regimen unable to achieve the goals related to optimal patient outcomes. In order to explore various ways to optimize the dosage of antibacterial drugs in ICU patients, novel dosing software using AI were developed to assist in the adjustment of antibiotic treatment, one of which was Bayesian forecasting. These plans can use the monitoring results of antibiotic treatment to further personalize the antibacterial program according to the clinical characteristics of each patient (39–42).

## Other Applications of AI in Sepsis

A study reported the practice results of using AI for quality improvement work. They introduced Sepsis Watch into the routine clinical care process, which is a sepsis detection and management platform based on deep learning (11). The purpose of Sepsis Watch is to improve the prediction and treatment of sepsis. It is formulated based on the quality improvement work report of a multidisciplinary team composed of statisticians, data scientists, data engineers and clinicians. The results show that it is feasible to integrate Sepsis Watch into routine clinical care, and the practice has also improved the implementation of local machine learning projects. Gonçalves et al. also reported the experience of applying AI algorithms in clinical practice, mainly introducing nurses' experience in early identification of sepsis through the use of technical tools developed by AI algorithms and its impact on the nursing work process (43). In the case introduced, the nurses participating in the process of technology integration can make rapid decisions in the early identification of sepsis.

Beginning in 2020, COVID-19 has spread all over the world, and infected patients have severe respiratory symptoms, and may have multiple complications such as severe acute respiratory syndrome, sepsis, septic shock and multiple organ failure. Effective ways to save cost and time are needed to mitigate the burden of disease. In order to seek potential treatments for COVID-19 among all existing drugs, a research combines systems biology and AI-based methods. By using the GUILDify v2.0 Web server as an alternative method, the effects of pirfenidone and melatonin on SARS-CoV-2 infection were confirmed. It also predicts the potential therapeutic effects of combination drugs on respiratory-related pathologies (44).

The pathogenic factors and processes of sepsis are complex and diverse. Its main feature is systemic inflammatory response. Severe Inflammatory Response Syndrome (SIRS) of non-infectious origin also has similar manifestations. Sepsis has a series of pathophysiological and genetic characteristics, which makes it difficult to distinguish from SIRS in clinical practice. This may be related to insufficient research on the key genes or pathways in the process of these diseases. Reasonable use of genetic biomarkers that are convenient for diagnostic tests/testing can make it possible to distinguish sepsis from SIRS. A team used previously published gene expression data sets, using two-tier gene screening, ANN data mining technology, and discovered biomarkers that can be used to identify and verify patients with SIRS, sepsis, and septic shock (45).

Causal AI can also be used to train and validate digital twin models, which can simulate critically ill patients and thus predict the response of sepsis patients to therapeutic interventions (46). The causal relationship between the organ system and a specific treatment is defined using a directed acyclic graph. The therapeutic effects and interactions of major organs at various stages are simulated using a hybrid method of agent-based modeling, discrete event simulation and Bayesian networks, which were visualized using relevant clinical markers. When the expected response simulated by the digital twin was compared with the actual patient response, it was found that the early treatment response of critical illness simulated by the AI model was very consistent with the patient's real response. The existence of a reliable digital twin model will allow clinicians to test the effects of interventions in a virtual environment before using them on real patients.

## The Safety and Challenges of Using AI in Sepsis

The potential of creating AI-based healthcare applications can match or exceed the ability of clinicians in specific diseases, such as sepsis. However, health care is a complex and ever-changing field with high requirements for safety. Any technical failure may cause harm to patients. When the AI system makes a decision, human clinicians and safety engineers essentially cannot control the process, and it is difficult to fully understand how the AI system accurately makes the decision. Compared with standard clinical practice, AI-based tools lack ethical constraints and safety regulations (47). The clinical setting of sepsis is very complex, and many variables (new therapies, new diagnoses, different intervention times and intervention methods) will affect the results. However, the requirements of all clinical settings shown in the computational model are difficult to achieve in the technical design stage (48). Therefore, the behavior of the software in the system may not adequately reflect appropriate clinical intentions. Currently, this problem is solved by ignoring some aspects of the process, such as by limiting the amount of information input, but it may lead to unintended consequences. One example is the loss of insensible fluid. It cannot be recorded electronically, which may cause the AI to prompt that more fluid needs to be refilled. However, in reality, the clinician sees that the patient has been waterlogged. In addition, when a machine interprets data, it cannot reason on the most important content like a human clinician. For example, clinicians may select to omit highly abnormal test results, which may be due to errors in sampling, testing, or recording.

In addition, there are problems in the AI model itself, for example, many studies have only trained and validated the model in the same patient cohort, but have not yet evaluated its generality to other populations. These models need to undergo further prospective testing to prove their benefits in clinical or other outcomes (49). AI will also face many implementation difficulties when used in clinical practice. Many organizations currently do not have sufficient conditions to implement AI in clinical practice, which requires considerable AI experts and mature information technology or IT capabilities, such as evaluation, merging, continuous monitoring, and recalibration of AI. The security and reliability of the collection and use of digital data also need to be addressed. Furthermore, most healthcare systems worldwide may not have enough capacity to successfully integrate AI into the current workflow. Decision-making and predictive models do not yet match the currently known healthcare systems, and a lot of improvements are needed to successfully integrate these innovations (50).

In short, there is still a big gap between the creation of AI algorithms and their implementation in clinical practice. AI cannot replace the clinical management role of experts in sepsis. AI-based algorithms should always be used as development tools until they can incorporate actions that are compatible with known physiology and prove that the results can be modified prospectively in multiple environments.

## Author Contributions

MW, XD, and JW carried out the concepts, design, definition of intellectual content, literature search, data acquisition, and manuscript preparation. MW carried out literature search, data acquisition, and manuscript editing. XD, JW, and RG performed manuscript review, including revision of key technical content and English expression. All authors have read and approved the content of the manuscript.

## Conflict of Interest

The authors declare that the research was conducted in the absence of any commercial or financial relationships that could be construed as a potential conflict of interest.
